# A Case of Acute Encephalopathy After the Initiation of FOLFOX Chemotherapy in a Patient With Colon Cancer

**DOI:** 10.7759/cureus.37237

**Published:** 2023-04-07

**Authors:** Jin S Kim, Ka U Lio, Hannah Henderson, Seyedmohammad Pourshahid

**Affiliations:** 1 Thoracic Medicine and Surgery, Temple University Hospital, Philadelphia, USA; 2 Internal Medicine, Temple University Hospital, Philadelphia, USA; 3 Neurology, Temple University Hospital, Philadelphia, USA; 4 Thoracic Medicine and Surgery, Lewis Katz School of Medicine, Temple University Hospital, Philadelphia, USA

**Keywords:** diagnosis of rare cases, posterior reversible encephalopathy syndrome (pres), hyperammonemia, encephalopathy, folfox

## Abstract

Acute encephalopathy is a change in the level of consciousness where the underlying etiology can be difficult to diagnose, and thus, difficult to treat, especially in the context of multi-organ diseases. We report a case of acute encephalopathy in a patient with end-stage renal disease (ESRD) on hemodialysis, chronic hypotension, and a recent diagnosis of colon cancer who presented shortly after initiation of FOLFOX, a chemotherapy regimen for treatment of colorectal cancer comprised of folinic acid (leucovorin), fluorouracil (5-FU), and oxaliplatin (eloxatin). We present a systematic approach to elucidate ambiguous causes of toxic-metabolic encephalopathy.

## Introduction

Acute encephalopathy describes a state of global disturbances in brain function with features that include a change in the level of consciousness, global alteration in cognition, acute onset, and an underlying structural or metabolic process [1]. “Acute” often refers to a rapidly developing pathobiological process in the brain over the course of fewer than four weeks, but usually within hours to a few days [2]. It may result from various insults, including primary neurologic conditions (i.e., hemorrhage, encephalitis, demyelinating diseases) or systemic conditions (i.e., infection, autoimmune, endocrine, metabolic disturbances, drug withdrawals, toxin ingestions). Diagnostic testing is vast and includes a multitude of tests such as cerebrospinal fluid (CSF) analyses, imaging, and advanced procedures to identify the etiology of encephalopathy. Even with treatment of the underlying condition, the clinical course and neurologic recovery may be protracted, particularly in older patients with a prior neurologic disease [3]. We report a case of acute encephalopathy in the context of multiple end-organ diseases shortly after the initiation of FOLFOX therapy.

## Case presentation

A 61-year-old woman with colon cancer presented as an ICU-level transfer from an outside hospital for acute encephalopathy. Her medical history included stage III colon cancer status post right-sided hemicolectomy, end-stage renal disease (ESRD) on hemodialysis, hypothyroidism, insulin-dependent diabetes mellitus, and chronic hypotension on midodrine with hemodialysis sessions. She was in her usual state of health. She had received her first dose of FOLFOX therapy at a 30% dose reduction. Then, she had her scheduled hemodialysis session the following day.

The next day, she was brought into the emergency department by emergency medical services for one day of somnolence and confusion. Vitals were notable for a temperature of 36.7 degrees Celsius, blood pressure 108/64, pulse 80/min, respiratory rate 22/min, and saturating 97% on 15L nonrebreather. Shortly thereafter, she was reported to be agitated and combative requiring chemical sedation. Then, she required intubation for airway protection and vasopressors for hypotension. Labs were remarkable for the following: sodium 133 (136-145 mmol/L), potassium 5.7 (3.5-5.1 mmol/L), bicarbonate 14 (21-32 mmol/L), anion gap 30, blood urea nitrogen 55 (6-20 mg/dL), creatinine 6.6 (0.55-1.3 mg/dL), phosphate 8.7 (2.5-5 mg/dL), calcium 8.4 (8.5-10.1 mg/dL), uric acid 9.1 (2.6-6 mg/dL), ammonia 50 (11-32 µmol/L), lactate 7.2 (0.4-2 mmol/L) and NT-pro-BNP >350,000 (5-125 pg/mL). Venous blood gas showed a pH of 7.16, partial CO_2_ 47 (41-51 mmHg), partial O_2_ 79 (25-40 mmHg), bicarbonate 16 (24-28 mmol/L), and O_2_ saturation 90% (40%-85%). She was admitted to the intensive care unit, received a hemodialysis session, and was pending further diagnostic testing by neurology. On hospital day 3, she was transferred from the outside hospital for further care. 

Upon transfer, she was found to be hemodynamically stable without the need for vasopressors. She was synchronous with the ventilator, on minimal ventilator support with spontaneous respirations, and had not received any sedatives after intubation. On physical exam, she had minimal response to external stimuli. Pupils were symmetric bilaterally with intact pupillary constriction and notable for the presence of roving eye movements. Her face was grossly symmetrical. Corneal, cough, oculocephalic, and gag reflexes were present. She had a flaccid tone in all extremities as well as absent reflexes. Non-purposeful, spontaneous movements of the upper extremities were present.

Pertinent laboratory results after transfer showed the following: sodium 135 (136-145 mmol/L), potassium 3.8 (3.5-5.1 mmol/L), bicarbonate 21 (21-32 mmol/L), anion gap 19, blood urea nitrogen 79 (6-20 mg/dL), creatinine 6.6 (0.55-1.3 mg/dL), phosphate 8.7 (2.5-5 mg/dL), calcium 7.6 (8.5-10.1 mg/dL), and ammonia 162 (15-45 µmol/L). Arterial blood gas showed a pH of 7.34, partial CO_2_ 40 (35-45 mmHg), partial O_2_ 101 (75-100 mmHg), bicarbonate 21 (20-28 mmol/L), and O2 saturation 96% (95%-98%). Infectious workup (urine, sputum, blood cultures), thyroid studies, folate, vitamin B12, thiamine, and liver function tests were unremarkable.

An initial non-contrast CT head revealed no intracranial abnormalities. Electroencephalogram (EEG) showed marked background slowing with intermittent attenuations suggestive of toxic metabolic encephalopathy. A lumbar puncture revealed a clear cerebrospinal fluid (CSF) with normal WBC counts and normal pressure. CSF studies were notable for elevated protein at 113 mg/dL and glucose at 115 mg/dL (Table 1).

**Table 1:** Cerebrospinal fluid cell count from tube 4 with actual results, normal range, and comments.

CSF (tube 4)	Actual	Normal range	Comments
Appearance	Hazy slight pink	Clear/ colorless	Bloody tap
Supernatant	Clear	Clear	Normal
WBC	3	0-5/ mm3	Normal
RBC	2040	0-10/mm3	Elevated
Glucose	115	40-70 mg/dL	Elevated
Protein	113	15-40 mg/dL	Elevated

**Figure 1:**
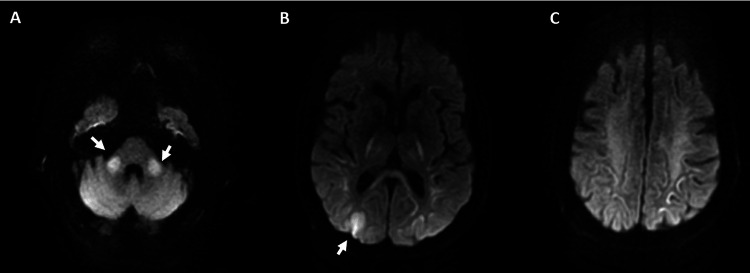
Brain MRI with and without contrast DWI axial images at time of presentation (A) Mild restricted diffusion within the middle cerebellar peduncles bilaterally; (B, C) Increased signal in the right parietal occipital hyperintensity and focal changes within thalami and corona radiata bilaterally. Most likely etiology is posterior circulation ischemia from a hypotensive event.

All other cerebral spinal fluid studies including infectious workup (i.e., gram stain, culture, varicella-zoster virus [VZV], herpes simplex virus [HSV], JC virus, Epstein Barr virus [EBV], enterovirus, cytomegalovirus [CMV], Lyme), autoimmune workup (i.e., oligoclonal IgG bands), and the paraneoplastic syndrome panel were negative. A brain MRI with and without contrast revealed bilateral FLAIR/T2-weighted hyperintensities in the brachium pontis, posterior limb of the internal capsules, and parietal occipital lobes concerning posterior circulation ischemia from a hypotensive event. The MRI also found areas of bilateral restricted diffusion on DWI/ACD sequences within the middle cerebellar peduncles, the posterior limbs of the internal capsules, the parietal occipital cortices, and the centrum semiovale. Mild diffuse dural thickening and enhancement were seen on T1 postcontrast sequences, which could be seen post-lumbar puncture or with underlying meningitis/meningoencephalitis. There were also background changes in white matter microvascular disease (Figures 1A, 1C).

The initial differentials in this patient were broad due to complex medical comorbidities and included infectious etiologies (i.e., bacterial/ viral meningoencephalitis), metabolic (i.e., uremia, hyperammonemia), paraneoplastic syndromes, nonconvulsive status epilepticus, brain metastasis, and posterior reversible encephalopathy syndrome (PRES).

The patient was started on empirical broad-spectrum treatment for bacterial and viral meningoencephalitis. She received daily hemodialysis sessions for potential uremic and hyperammonemic encephalopathy. She also received lactulose, rifaximin, and an aggressive bowel regimen. After 3-4 days of therapy (and 6-7 days of total hospitalization), the patient had significant improvement in mentation with spontaneous eye opening and was able to follow simple commands. She was successfully liberated from the ventilator and transferred to the medical floor the following week. Throughout the hospitalization, her blood pressure varied from 70/50s to 140/90s. She was started on florinef for chronic hypotension noted to be refractory to midodrine for multisystem atrophy. She was discharged to home without focal neurologic deficits.

**Figure 2:**
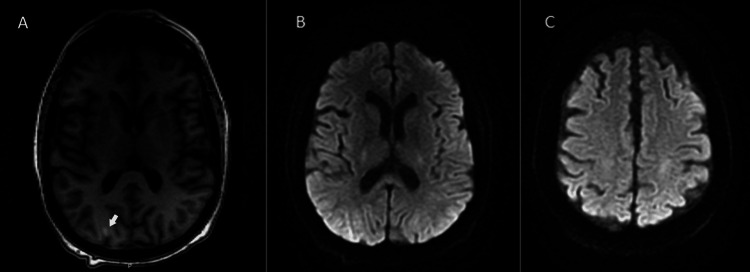
Brain MRI with and without contrast DWI axial images one week after prior imaging (A) New T1 gyriform cortical enhancement within the right occipital lobe corresponding to the area of DWI diffusion restriction found in Figure 1B; (B, C) interval resolution of hyperintensities previously seen in Figures 1B, 1C.

A repeat brain MRI showed new T1-sequence gyriform cortical hyperintensity within the right occipital lobe compatible with cortical laminar necrosis secondary to ischemia with evidence of left-sided reperfusion. There was a resolution of the restricted diffusion within the left parieto-occipital lobe, bilateral internal capsules, and cerebral peduncles (Figures 2A-2C).

## Discussion

The presence of complex comorbidities including ESRD (chronic inflammatory state, uremia), chronic hypotension (hypoperfusion state), hypothyroidism, diabetes (fluctuating glucose level), and malignancy could all be potential triggers leading to an acute derangement in consciousness and cognition. In addition, recent initiation of chemotherapy and sedative/analgesic agents received pre- and post-intubation could also be possible triggers leading to encephalopathy. In this case, we believe the cause of acute encephalopathy was likely the result of a culmination of etiologies including FOLFOX-induced hyperammonemia, PRES, and chronic hypotension. In the following paragraphs, we present our clinical approach to elucidating the probable cause(s) of encephalopathy.

First, her initial CT head without contrast did not reveal any abnormalities, eliminating any acute primary structural insults such as intracranial hemorrhage, traumatic brain injury, large intracranial lesions, or normal pressure hydrocephalus. Her EEG results did not reveal any focal epileptic discharges suggestive of seizure but indicated toxic-metabolic etiologies.

Initial blood testing was notable for elevated BUN but unsurprising given her history of ERSD. The presence of uremic encephalopathy generally parallels the severity of azotemia in acute kidney injury but does not necessarily correlate with chronic renal failure. Stable uremic encephalopathy may present with a fine tremor, asterixis, and hyperreflexia, whereas in advanced uremic encephalopathy, anorexia, myoclonus, upper motor neuron changes, gait/speech changes, and coma can be observed.

FOLFOX is a combination chemotherapy agent that includes 5-Fluorouracil (5-FU), Oxaliplatin, and Leucovorin used to treat gastrointestinal cancers. FOLFOX-induced hyperammonemic encephalopathy has been previously described but is limited to case reports. A wide array of clinical presentations with varying severity and acuity has been reported, and the onset of symptoms after the start of chemotherapy typically was subacute, ranging from nine to 23 days [4-6].

Ammonia levels greater than 100-150 µmol/L are suggestive of hyperammonemia such as in this patient’s case. Hyperammonemia is a toxic accumulation of ammonia that is shunted into the systemic circulation. As ammonia levels rise, it crosses the blood-brain barrier, converting into glutamine, and increasing cerebral volume via hyperosmotic effects. This cross-over then can cause cerebral edema and brain herniation [7]. Patients at risk are those with hepatic disease, in-born metabolic disease, a history of lung transplantation, and valproic acid therapy [8]. Once confirmed, it is important to identify the trigger for hyperammonemia including hypovolemia, gastrointestinal bleeding, hepatic disease, hepatoportal shunt, drugs, or infection. Important to note, levels can be falsely elevated due to hemolysis with delayed processing and exposure to room temperature. Venous ammonia concentration may not be accurate and could be influenced by many factors (i.e., the sample not being placed on ice and the use of a tourniquet) [9,10].

Two hypotheses on the mechanism of 5-FU-induced hyperammonemia have been proposed. The first hypothesis is the presence of fluoroacetate, an intermediate product of 5-FU, inhibiting the Krebs cycle and leading to a deficiency of adenosine triphosphate (ATP), which leads to a delay in the metabolism of ammonia [11]. The second hypothesis is the deficiency of dyropyrimidine dehydrogenase (DPD), which is an enzyme responsible for metabolizing 5-FU. A deficiency of DPD can lead to the accumulation of 5-FU and an increase in ammonia [12]. Patents with FOLFOX-induced hyperammonemic encephalopathy often present with a delay of two days after infusion, and commonly present with cognitive impairment, seizures, and confusion [13,14]. Several factors associated with hyperammonemia have been reported, including renal failure, dehydration, sarcopenia, and infection [15]. In a large observational study of 74 patients, Okamato et al. found that CKD stage G3 or higher (eGFR <60 mL/min/1.73m^2^) is a risk factor for developing hyperammonemia during FOLFOX therapy [16]. Currently, there is no specific treatment for 5-FU-induced hyperammonemic syndrome other than supportive therapies including ammonia-lowering medications and chelators.

Regarding infectious evaluation, blood cultures were negative. The patient was anuric and unable to provide a urine specimen. She did not have any episodes of fever, leukocytosis, or leukopenia. Due to her underlying malignancy and the meningeal enhancement on the brain MRI (albeit after the initial lumbar puncture attempt), encephalitis (infectious, paraneoplastic, or autoimmune) could not be ruled out. Lumbar puncture was reattempted, and results were negative for bacterial, paraneoplastic, or autoimmune causes.

Another differential diagnosis to consider is acute toxic leukoencephalopathy which can be seen in cases of polysubstance abuse, lead toxicity, carbon monoxide poisoning, inhalant use, immunosuppression, chemotherapy, and intracranial irradiation [17]. It can cause white matter changes on brain MRI related to chronic exposure which was absent in this patient's history.

Interestingly, the brain MRI did show abnormal signal changes in the posterior circulation concerning ischemia from hypotension with partial reversal on the subsequent brain MRI. Posterior reversible encephalopathy (PRES) was first described in 1996 in a case series of 15 patients with the reversible syndrome of headache, altered mental status, and seizures associated with findings indicative of predominantly posterior leukoencephalopathy on imaging studies [18]. PRES is increasingly recognized and reported, however, the incidence is not well known. It is a neurologic syndrome defined by both clinical and radiographic features and commonly associated with those with renal disease, hypertension, use of cytotoxins, or immunosuppressive therapy. The exact pathophysiology has not been completely explained but a well-recognized mechanism is the presence of cerebral autoregulatory failure, hypertension, and endothelial dysfunction causing brain capillary leakage, blood-brain barrier breakdown, and extravasation of cerebral fluid into the brain parenchyma [18-20].

## Conclusions

Acute encephalopathy is a common presentation in critically ill patients with multiple comorbidities. Differential diagnoses are broad, and identifying the exact etiology may be difficult. We present a rare case of acute encephalopathy after FOLFOX-induced hyperammonemia with subsequent development of PRES in a patient with end-stage disease. Early identification of the underlying etiology triggering acute encephalopathy is crucial in preventing future episodes, understanding clinical course, and discussing prognosis.
